# Tolerability and efficacy of gamma knife radiosurgery on hepatocellular carcinoma with portal vein tumor thrombosis

**DOI:** 10.18632/oncotarget.6118

**Published:** 2015-10-14

**Authors:** Xiao-Jie Lu, Jing Dong, Li-Juan Ji, Li-Xin Xiao, Chang-Quan Ling, Jun Zhou

**Affiliations:** ^1^ Department of Gastroenterology, Shanghai East Hospital, Tongji University School of Medicine, Shanghai, China; ^2^ Outpatient Department, Zhejiang Provincial People's Hospital, Hangzhou, Zhejiang Province, China; ^3^ Department of Rehabilitation, The Affiliated Huai'an Hospital of Xuzhou Medical College and The Second People's Hospital of Huai'an, Huai'an, China; ^4^ Department of Gamma Knife, The 411st Hospital of Chinese People's Liberation Army, Shanghai, China; ^5^ Changhai Hospital of Traditional Chinese Medicine, Second Military Medical University, Shanghai, China; ^6^ Department of Oncology and Hematology, Shanghai East Hospital, Tongji University School of Medicine, Shanghai, China

**Keywords:** hepatocellular carcinoma, portal vein tumor thrombosis, gamma knife, adverse events, overall survival

## Abstract

This is a retrospective study on the safety and efficacy of gamma knife radiosurgery (GKR) in treating hepatocellular carcinoma (HCC) with portal vein tumor thrombosis (PVTT). Patients with confirmed HCC and PVTT were allocated into two groups based on the treatments they received (palliative or GKR). A total of 138 patients were included (74 in the palliative group, 64 in GKR group). No significant differences in baseline characteristics existed between the two groups. Treatment-related adverse events (AEs) were recorded and compared between groups. The majority of AEs were mild to moderate and subsided naturally or after medication. There was no AE-induced death. The influences of baseline characteristics and treatment options on patients' OS were analyzed. The median OS of patients in the palliative and GKR group were 3.0 months (95% CI: 2.719-3.281) and 6.1 months (95% CI: 4.706-7.494) respectively (*p* = 0.003). Multivariate analysis revealed that GKR treatment, performance status 0-1, Child A, smaller tumor diameter and monolobar distribution were significant favorable prognosticators. Subgroup analyses showed OS benefit of GKR regardless of PVTT location (main or branch of PVTT). In conclusion, GKR is well tolerated in selected HCC-PVTT patients and can confer OS benefit, which needs validation in future prospective studies.

## INTRODUCTION

Hepatocellular carcinoma (HCC) represents the second leading cause of cancer death worldwide, with half of the cases being in China [1]. It was reported that approximately 10%-40% of HCC patients are diagnosed with portal vein tumor thrombus (PVTT) [2, 3] and that up to 44% of HCC patients are complicated with PVTT at the time of death [4]. According to the Barcelona Clinic Liver Cancer (BCLC) classification system [5], HCC patients with PVTT are classified as advanced stage (or Stage C) and bear a rather dismal prognosis with expected median survival span of about only 2.7-3.0 months [3, 6].

Sorafenib is currently regarded as the standard treatment for HCC-PVTT patients [7, 8]. However, clinical trials [9, 10] showed that it can only produce 2-to-3 months of overall survival (OS) benefit in BCLC stage C patients. Moreover, the high price of sorafenib makes it unaffordable for many patients in the developing countries such as China. Therefore, there is an urgent need to explore alternative therapeutics for HCC patients with PVTT.

Radiotherapies have long shown promise in treating HCC patient with PVTT, such as the intraarterial injection of ^131^I-labeled-iodized oil [11] and the ^90^Y-based radioembolization [12]. Gamma knife radiosurgery (GKR) is a kind of external radiation therapy that have been reported to show favorable effects in treating brain metastases of HCC in retrospective studies [13, 14]. In a few cancer centers in China, it has been practiced for treating HCC-PVTT patients for more than ten years. Recently, our team [15] conducted a retrospective study which found that the median OS of HCC-PVTT patients receiving combined therapy of transarterial chemoembolization (TACE) and GKR was significantly longer than that of HCC-PVTT patients receiving TACE alone. However, it is still not clear whether patients receiving combined therapy lived longer as a result of therapeutic effects or whether they received more therapeutics simply because they lived longer (because of a more benign course). And it is not clear either whether the observed OS gain in the TACE plus GKR group versus TACE alone group was owing to GKR treatment per se or owing to the synergic effects of TACE and GKR. To address these questions, we conducted a separate retrospective study to investigate the safety and efficacy of GKR monotherapy on HCC patients with PVTT.

## RESULTS

### Baseline characteristics

**Figure 1:**
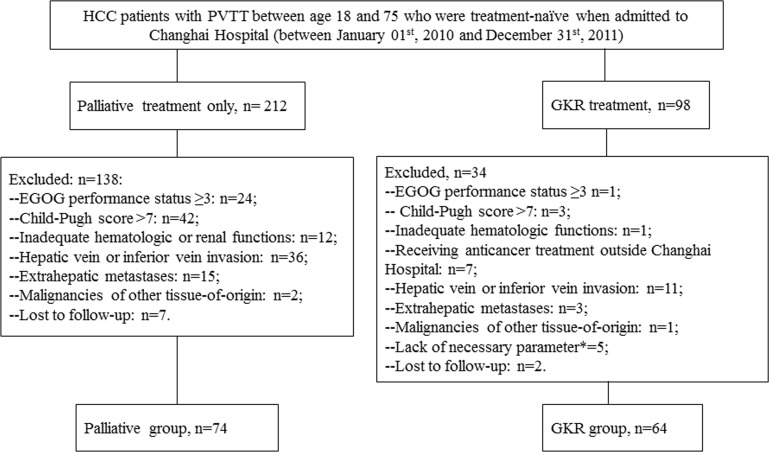
Flowchart of patients inclusion and exclusion *: Referring to patients whose clinical/laboratory follow-ups were so incomplete that subsequent analyses were impossible.

During the study period, a total of 310 HCC patients with confirmed PVTT were admitted to Changhai Hospital (Shanghai, China). According to the patient inclusion and exclusion criteria, 138 patients were included into this study finally, with 74 in the palliative group and the other 64 in the GKR group (Figure 1). No significant differences in baseline characteristics existed between the two groups (Table 1).

**Table 1:** Baseline characteristics of patients included in this study

Baseline characteristics		Palliative group (n=74)	GKR group (n=64)	*P* value*
Age	Mean±SD	54.34±11.01	52.83±9.53	0.394
Gender	Male (n=117)	64	53	0.549
	Female (n=21)	10	11	
Etiologies	HBV (n=116)	61	55	0.722
	HCV (n=7)	4	3	
	Alcohol(n=10)	5	5	
	Others(n=5)	4	1	
Type of PVTT	Branches of portal vein (n=71)	35	36	0.294
	Main portal vein (n=67)	39	28	
Cirrhosis	Absent (14)	9	5	0.573
	Precent (124)	65	59	
Child–Pugh	A (n=107)	54	53	0.167
	B7 (n=31)	20	11	
ECOG PST	0-1 (n=99)	49	50	0.121
	2 (n=39)	25	14	
Tumor nodules	Single (n=65)	31	34	0.187
	Multiple (n=73)	43	30	
Largest tumor size	Mean±SD	9.79±4.77	8.14±3.73	0.261
	≤5cm	15	11	0.098
	5-10cm	25	33	
	>10cm	34	20	
Bilobar	No (n=83)	41	42	0.221
	Yes (n=55)	33	22	
AFP	≤400 ng/ml (n=49)	25	24	0.649
	>400 ng/ml (n=89)	49	40	
Total bilirubin	Mean ±SD	1.12±0.64	1.10±0.58	0.552
	>1.5 mg/dl	15	17	0.302
Albumin	Mean ±SD	34.77±4.11	35.58±4.22	0.258
	<30 g/L	13	9	0.349
ALT	Mean ±SD	44.51±19.67	43.55±21.51	0.274
	>40 u/L	15	13	0.995
AST	Mean ±SD	46.27±20.80	42.13±21.10	0.248
	>40 u/L	17	11	0.399
GTT	Mean ±SD	178.22±132.03	182.31±158.86	0.869
	>50 u/L	47	36	0.385
INR	Mean ±SD	1.03±0.19	1.06±0.21	0.493
	≥1.3	5	5	0.811
WBC	Mean ±SD	5.26±1.04	5.55±1.14	0.119
	<4.0×109/L	5	6	0.571
HB	Mean ±SD	123.9±12.02	125.70±17.26	0.578
	<110 g/L	8	5	0.548
Platelets	Mean ±SD	110.2±17.59	112.05±19.66	0.562
	≤100×10^9^/L	21	19	0.866

*: Intergroup differences in baseline characteristics were analyzed using t-test for continuous data and Chi-Square test or Fisher exact test (for small samples) for categorical variables. *P*< 0.05 was considered statistically significant. TACE: transarterial chemoembolization; GKR: gamma knife radiosurgery; PVTT: portal vein tumor thrombosis; HBV: hepatitis B virus; HCV: hepatitis C virus; ECOG PST: Eastern Cooperative Oncology Group Performance status; INR: international normalized ratio; AFP: alpha fetal protein; ALT: alanine transaminase; AST: aspartate transaminase; WBC: white blood cell; Hb: hemoglobin; GGT: gamma-glutamyl transpeptidase.

### Safety and procedure-related adverse events

As mentioned above, clinical follow-ups (blood tests, physical examinations, etc.) in the palliative group were not as regular as in the GKR group. So, AEs data of the palliative group were incomplete. Nausea/vomiting, fatigue, abdominal pain, anorexia, radiodermatitis and transient liver function impairment were common GKR-related AEs (Tables 2 and 3). The majority of them, however, were mild-to-moderate (grade 1-2) ones and subsided naturally or after medication. Compared with pre-procedure, there were significantly more cases of liver function impairment in 3 months post-procedure (evidenced by elevations in alanine transaminase [ALT], aspartate transaminase [AST], total bilirubin [TB] and gamma-glutamyl transpeptidase [GTT]) (Table 3). No deaths within 4 weeks post-procedure were attributable to GKR-related AEs.

**Table 2:** Main procedure-related clinical adverse events by CTCAE grades

CTCAE	Grade 1	Grade 2	Grade 3	Grade 4	All grades
Abdominal pain	12(18.75)	9(14.06)	2(3.13)	0	21(32.8)
Anorexia	8(12.5)	16(25)	0	0	24(37.5)
Ascites	1(1.56)	0	0	0	1(1.56)
Constipation	2(3.13)	2(3.13)	0	0	4(6.25)
Fatigue	8(13)	5(8)	0	0	13(20)
Nausea/vomiting	11(17.19)	15(23.44)	0	0	26(40.63)
Pneumonitis	0	2(3)	0	0	2(3)
Dermatitis	11(17.19)	5(7.81)	0.00	0.00	16(25)

CTCAE: National Cancer Institute Common Terminology Criteria for Adverse Events.

**Table 3:** Main procedure-related laboratory adverse events by CTCAE grades

CTCAE	pre-procedure		3 months post-procedure		*p* value*	*p* value#
	All grades	Grade≥3	All grades	Grade≥3		
Leukocytopenia	6(9.4)	0	9(14.6)	3(4.7)	0.375	0.25
HB	5(7.8)	0	3(4.7)	0	0.5	1
Thrombocytopenia	19(29.7)	0	27(42.2)	3(4.7)	0.077	0.25
Hypoalbumin	9(14.1)	0	13(20.3)	0	0.344	1
ALT/AST↑	24(37.5)	0	39(60.9)	8(12.5)	0.017	0.008
TB↑	17(26.6)	0	33(51.6)	3(4.7)	0.002	0.25
GTT↑	36(56.3)	0	39(60.9)	17(26.6)	0.664	<0.001
Creatinine	0	0	2(3.1)	0	0.5	1
INR	5(7.8)	0	11(17.2)	0	0.109	1

*: Intragroup differences in all grades LAEs between baseline and month 3 were analyzed with Exact McNemar's test, *p*<0.05 was considered statistically significant. #: Intragroup differences in grade≥3 LAEs between baseline and month 3 were also analyzed with Exact McNemar's test. TB: total bilirubin; ALT: alanine transaminase; AST: aspartate transaminase; INR: international normalized ratio; GGT: gamma-glutamyl transpeptidase; CTCAE: National Cancer Institute Common Terminology Criteria for Adverse Events.

### Survival analyses

**Figure 2:**
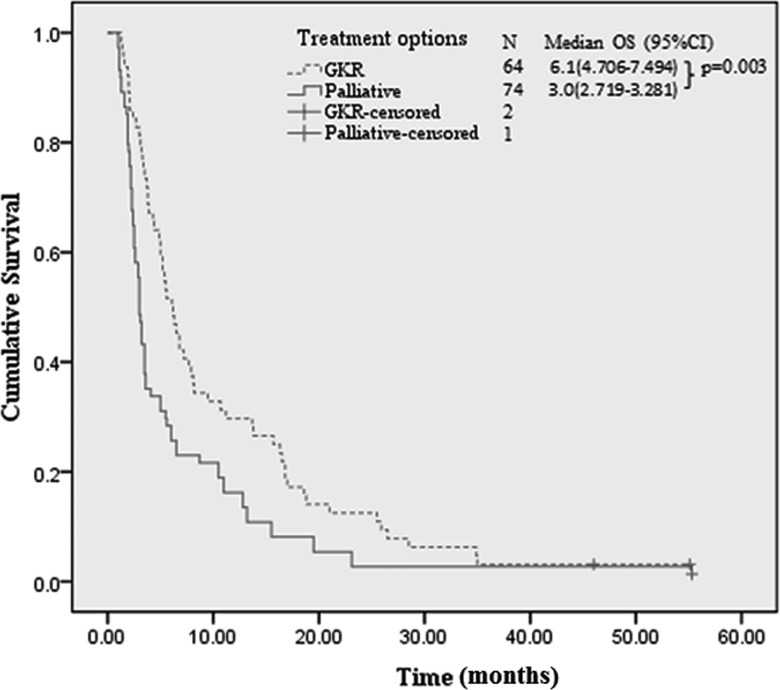
Kaplan-Meier survival curves of patients by treatment options GKR: gamma knife radiosurgery.

Only 3 patients were still alive by the last follow-up (December 20^th^ 2014). The median overall survival (OS) of patients in the palliative and GKR group were 3.0 months (95% CI: 2.719-3.281) and 6.1 months (95% CI: 4.706-7.494) respectively (*p* = 0.003) (Figure 2, Table 4). On multivariate analysis (Table 4), GKR showed significant survival benefit (hazards ratio [HR], 0.538; 95%CI, 0.356-0.814; *p* < 0.001) versus palliative treatment. Besides GKR treatment, PST 0-1 (HR: 0.495; 95%CI: 0.267-0.796; *p* < 0.001), Child A (HR: 0.534; 95%CI: 0.333-0.857; *p* < 0.001), tumor diameter ≤5 cm (HR: 0.452; 95%CI: 0.258-0.794; *p* <0.001) and monolobar distribution (HR: 0.584; 95%CI: 0.383-0.892; *p* < 0.001) were significant favorable prognosticators of OS relative to PST 2, Child B7, tumor diameter > 10 cm and bilobar distribution, respectively (Table 4). Subgroup (stratified by PVTT location) multivariate analyses showed that GKR produced survival benefits both in patients with branch of PVTT (HR: 0.634; 95% CI: 0.393-0.961; *p* = 0.041) and in patients with main PVTT (HR: 0.389; 95%CI: 0.227-0.561; *p* = 0.018).

**Table 4:** Univariate and multivariate survival analyses by baseline characteristics and treatment options

		Univariate analysis		Multivariate analysis	
		mOS (95% CI), months	*P* value	Hazard ratio (95% CI)	*P* value
All patients		3.9(2.931-4.869)			
Age	<50 (n=50)	3.6(2.677-4.523)	0.45		
	≥50 (n=88)	4.1(2.445-5.755)			
Gender	male (n=117)	3.7(2.807-4.593)	0.04	1	
	female (n=21)	6.8(4.987-8.597)		0.765(0.438-1.335)	0.35
Etiologies	HBV (n=116)	3.6(2.578-4.812)	0.46		
	HCV (n=7)	3.7(2.642-4.953)			
	alcohol(n=10)	4.0(2.982-4.893)			
	others(n=5)	4.0(2.886-4.965)			
Type of PVTT	branches of portal vein (n=71)	6(4.761-7.239)	<0.001	0.887(0.573-1.372)	0.6
	main portal vein (n=67)	3.2(2.901-3.499)		1	
Cirrhosis	absent (14)	4.5(3.391-5.456)			
	precent (124)	3.4(2.333-4.456)	0.16		
Child–Pugh score	A (n=107)	5.2(4.186-6.214)	<0.001	0.534(0.333-0.857)	<0.001
	B7 (n=31)	2.4(2.931-4.869)		1	
ECOG PST	0-1 (n=99)	5.9(4.526-7.223)	<0.001	0.495(0.267-0.796)	<0.001
	2 (n=39)	3.1(2.923-3.479)		1	
Tumor nodules	single (n=65)	6.6(3.225-8.987)		1	
	multiple (n=73)	3.5(2.945-5.003)	0.04	1.202(0.903-1.406)	0.1
Tumor diameter	≤5cm (n=26)	5.5(0.003-10.997)	<0.001	0.452(0.258-0.794)	<0.001
	5-10cm (n=58)	6.2(5.240-7.16)		0.586(0.373-0.920)	
	>10cm (n=54)	2.4(1.886-2.914)		1	
Tumor distribution	Monolobar (n=83)	5.6(4.265-6.935)	<0.001	0.584(0.383-0.892)	<0.001
	Bilobar (n=55)	3(2.318-3.682)		1	
AFP	≤400 ng/ml (n=49)	5.4(4.6-6.2)	0.09	0.672(0.429-1.051)	0.1
	>400 ng/ml (n=89)	3.5(2.853-4.147)		1	
Treatment option	Palliative (n=74)	3.0(2.719-3.281)	0.003	1	
	GKR (n=64)	6.1(4.706-7.494)		0.538(0.356-0.814)	<0.001

***P*** < 0.05 was considered statistically significant. All statistical analyses were conducted using SPSS 17.0 (SPSS 17.0 for Windows, SPSS, Chicago, III). GKR: gamma knife radiosurgery; PVTT: portal vein tumor thrombosis; PV: portal vein; HBV: hepatitis B virus; HCV: hepatitis C virus; ECOG: Eastern Cooperative Oncology Group; INR: international normalized ratio; AFP: alpha fetal protein.

Multivariate analysis in the GKR group showed that Child A (HR: 0.386; 95% CI: 0.196-0.758; *p* = 0.006), PST 0-1 (HR: 0.412; 95% CI: 0.209-0.791; *p* = 0.014) and AFP≤400 (HR: 0.477; 95% CI: 0.276-0.823; *p* = 0.008) were significant favorable prognosticators of OS relative to Child B7, PST 2 and AFP >400, respectively. (Supplementary table 1).

## DISCUSSION

The treatment of HCC patients complicated with PVTT has long been a crux in the clinic. In this retrospective study, we for the first time investigate the safety and efficiency of GKR monotherapy in treating HCC-PVTT patients.

Although a substantial part of patients receiving GKR treatment experienced procedure-related AEs (Tables 2 and 3), these AEs were predominantly mild to moderate and were easily controlled with medication or subsided naturally. Only a small proportion of patients experienced severe AEs and none of them led to patient death. These results indicate that GKR can be well tolerated in selected HCC-PVTT patients as in our study.

Survival analyses revealed that GKR provided a 3.1-month survival benefit relative to palliative treatment (Figure 2, Table 4), which is comparable to the effect of sorafenib in advanced HCC patients [9, 10]. Moreover, GKR showed OS benefit in both patients with main PVTT and patients with branch of PVTT. Besides treatment option, factors that impacted OS in our study included ECOG PST, Child score and tumor characteristics (size and distribution), which is in line with the results of many similar studies [17-19]. Interestingly, patients with branch of PVTT had significantly longer median OS compared to patients with main PVTT upon univariate analysis (6 versus 3.2 months, *p* < 0.001), whereas on multivariate analysis, PVTT location has no significant impact on OS (HR: 0.887; 95%CI: 0.573-1.372; *p* = 0.6). Logistic regression revealed that the presence of main PVTT was significantly correlated with larger tumor size and bilobar tumor distribution (data not shown), indicating that it is larger tumor size and bilobar tumor distribution that rendered patients more prone to developing main PVTT and bearing a worse prognosis.

Although this study is retrospective in nature, which is its main limitation, it possesses several strengths. Firstly, as Changhai Hospital (Shanghai, China) is one of the best liver cancer treatment centers in China, we have enough patients meeting the including criteria. The total number of patients enrolled in this study was more than one hundred and there was no significant difference in baseline characteristics between the two groups. Secondly, we have a palliative group as a control comprising more than 70 patients who received palliative treatment only. We have complete data on their OS though their AEs data were incomplete. This group served as the basis for OS benefit analyses of GKR treatment. And thirdly, the follow-up period of this study spanned 4 years (from January 01^st^, 2010, the initiation of this study, to December 20^th^ 2014, the last follow-up) and the vast majority of participants were followed up successfully (Figure 1). Given that the treatment allocation of HCC with PVTT is influenced by many factors such as patients' will, general condition, liver function, tumor status, economic status and complicated diseases, it is rather challenging to conduct a randomized perspective clinical trial on this regard. Therefore, this retrospective study provides valuable information on the safety and efficacy of GKR monotherapy in treating HCC-PVTT.

Another limitation of this study is the lack a sorafenib-treatment only group to enable direct comparison of sorafenib and GKR. Recent studies [20-25] have investigated the efficacy of sorafenib combined with other therapeutic strategies such as TACE, radiofrequency ablation and radiotherapy on HCC-PVTT patients. It is meaningful to conduct similar studies in the future to compare GKR with sorafenib or to explore whether the two therapies can be used concomitantly.

In conclusion, this retrospective study provides the first evidence of the tolerability and efficacy of GKR monotherapy in treating HCC with PVTT, which has been practiced in China for several years. Our results indicate that GKR monotherapy are generally well tolerated and can confer survival gain in selected HCC-PVTT patients as in our study. Future perspective studies are needed to validate these results and to determine whether GKR can be recommended as a therapeutic option for HCC-PVTT patients.

## MATERIALS AND METHODS

### Study design and patents enrollment

Institutional Ethics Committee of Changhai Hospital (Shanghai, China) approved this retrospective study. The diagnoses of HCC were confirmed either by typical imaging manifestations or by biopsy according to recommendations by the European Association For The Study Of The Liver (EASL) [8]. PVTT was confirmed by the presence of intraluminal filling defect with an enhancement pattern similar to that of HCC on contrast-enhanced computed tomography (CT) and/or magnetic resonance imaging (MRI).

The medical records of HCC patients admitted to Changhai Hospital (Shanghai, China) between January 01^st^, 2010 and December 31^st^, 2011 were reviewed. Patients inclusion criteria: 1) HCC patients with confirmed PVTT between age 18 and 75 who were treatment-naïve when admitted to Changhai Hospital; 2) ECOG (Eastern Cooperative Oncology Group) performance status (PST): 0-2; 3) Child-Pugh score 5-7; 4) Adequate hematologic (granulocyte count > 1.5×10^9^/L, platelets > 50×10^9^/L) and renal (creatinine <2.0 mg/dL) functions; 5) Received palliative treatment alone or in combination with GKR. Patients exclusion criteria:1) Patients who received anticancer treatment other than GKR, such as hepatic resection, sorafenib, radiofrequency ablation and TACE, or anticancer treatment outside Changhai Hospital (Shanghai, China); 2) Patients with hepatic vein or inferior vein invasion, extrahepatic metastases, or malignancies of other tissue-of-origin; 3) Patients with signs of decompensated cirrhosis such as clinical hepatic encephalopathy and refractory ascites; 4) Lack of necessary parameters for subsequent analyses; 5) Patients who received more than one episode of GKR.

### Treatment procedures

For each patient, treatments were allocated based upon patient will and clinicopathological characteristics, which were assessed by the HCC Expert Team in Changhai Hospital. This team comprised oncologists, hepatologists, interventional radiologists and radiation oncologists. Written informed consent was obtained from each patient prior to GKR.

Palliative treatments referred to symptomatic treatment or supportive care that aimed mainly at alleviating patients' symptoms and improving their quality of life. Patients who received palliative treatments only were allocated to palliative group. It should be noticed that patients in the GKR group also received palliative treatment.

GKR procedure: Patient was immobilized with vacuum bags in the supine position with the arms raised above the head during simulation. GTV was delineated by contrast-enhanced MRI or CT scan, which included both tumor thrombosis and the primary tumor in the liver. If the primary tumor in the liver was close to tumor thrombosis, they were included into one entire target region. If the primary lesion was far away from the tumor thrombosis or if there were multiple lesions, they were then included into different target regions. The planning target volume (PTV) was defined as a 5-10 mm margin around the gross tumor volume (GTV) with the aid of Treatment Planning System (TPS, OUR New Medical Technologies Co. Ltd., Shenzhen, China). The median tumor margin dose was 40 Gy (ranging from 35 to 45 Gy), with a median isodose line of 55% (50%-60%). The dose prescription was limited by adjacent normal tissue tolerances and the volume of liver that could be spared (at least 1/3 of the liver volume should be spared). In brief, the liver and adjacent normal tissues (for example, gastrointestinal tract, kidney and spinal cords) were delineated during the target planning process. Dose-volume histograms were harnessed to ensure that normal tissue tolerances were not exceeded. GKS was performed with Gamma Master Space Body Knife System (also called OUR-QGD system, OUR New Medical Technologies Co. Ltd., Shenzhen, China), which is a stereotactic body radiotherapy system. The total dose of gamma knife radiotherapy was carried out in 10-12 days (five consecutive days per week).

### Patient follow-up and data collection

According to the institutional protocol, regular clinical follow-ups are mandatory for all HCC patients admitted to Changhai Hospital. The vast majority of patients in the GKR groups adhered to this policy, whereas a substantial proportion of patients in the palliative group failed to do so. Clinical follow-ups were carried out 1 month after GKR and every 2 months thereafter, including detailed history and physical examinations, contrast-enhanced CT or MRI, and a complete panel of blood chemistry. GKR-related adverse events (AEs) were recorded until 3 months post-GKR according to National Cancer Institute Common Terminology Criteria for Adverse Events (CTCAE) v3.0 [16]. Survival was calculated from the day of the baseline evaluation to the day of death (confirmed by medical records or by family members).

### Statistical analyses

Intergroup differences in baseline characteristics were analyzed using t-test for continuous data and Chi-Square test or Fisher exact test (for small samples) for categorical variables. Intragroup differences in LAEs between baseline and month 3 were analyzed with Exact McNemar's test. Median overall survival (mOS) was calculated using the Kaplan-Meier method and compared by the log-rank test. Variables with *P* values < 0.25 on univariate analyses were included in multivariate analysis (Cox proportional hazards model). *P* < 0.05 was considered statistically significant.

## SUPPLEMENTARY MATERIAL TABLE


