# Supplementary material for: Tolerability and efficacy of gamma knife radiosurgery on hepatocellular carcinoma with portal vein tumor thrombosis
Source: Oncotarget. 2015 Oct 14;7(3):3614–22. doi: 10.18632/oncotarget.6118 (PMC4823131; doi:10.18632/oncotarget.6118)
Supplement: Supplementary file 1 [file oncotarget-07-3614-s001.pdf]

Tolerability and efficacy of gamma knife radiosurgery on hepatocellular carcinoma with portal vein tumor thrombosis

Supplementary Material

Supplementary Table 1: Univariate and multivariate analysis of overall survival in the GKR group by baseline characteristics.

		Univariate analysis		Multivariate analysis	
		mOS (95% CI), months	*P* value	Hazard ratio (95% CI)	*P* value
All patients in the GKR group (n=64)		6.1(4.706-7.494)			-
Age	<50 (n=21)	6.5(5.154-7.846)	0.769		-
	≥50 (n=43)	5.5(4.108-6.892)			-
Gender	male (n=53)	5.6(4.304-6.896)	0.669		-
	female (n=11)	6.5(3.445-9.555)			-
Etiologies	HBV (n=55)	6.1(4.145-8.279)	0.338		-
	HCV (n=3)	7.1(5.682-9.159)			-
	alcohol(n=5)	6.5(5.184-8.156)			-
	others(n=1)	4.5			-
Type of PVTT	branch of PV (n=36)	6.3(3.948-8.652)	0.172	0.674(0.397-1.14)	0.145
	main PV (n=28)	5.0(2.666-7.334)			-
Cirrhosis	absent (5)	7.4(5.127-9.236)	0.276		-
	precent (59)	6.0(4.174-8.191)			-
Child–Pugh score	A (n=53)	6.5(4.870-8.130)	0.009	0.386(0.196-0.758)	0.006
	B7 (n=11)	3.0(1.921-4.079)		1	-
ECOG PST	0-1 (n=50)	7.1(5.146-8.991)	0.015	0.412(0.209-0.791)	0.014
	2 (n=14)	4.0(2.935-5.011)		1	-
Tumor nodules	single (n=34)	8.0(5.123-11.014)	0.15	1	-
	multiple (n=30)	4.1(3.015-5.373)		1.231(0.911-1.439	0.092
Largest tumor size	≤5cm (n=11)	7.7(4.463-10.937)	0.091	0.945(0.429-2.084)	0.803
	5-10cm (n=33)	6.8(5.679-7.921)		0.811(0.420-1.564)	-
	>10cm (n=20)	3.1(0.470-5.370)		1	-
Tumor distribution	monolobar (n=42)	6.3(4.489-8.111)	0.315		-
	bilobar (n=22)	5.5(3.344-7.656)			-
AFP	≤400 ng/ml (n=24)	7.9(1.621-16.234)	0.012	0.477(0.276-0.823)	0.008
	>400 ng/ml (n=40)	5.2(2.569-7.831)		1	-

Median overall survival (mOS) was calculated using the Kaplan-Meier method and compared by the log-rank test. Variables with P values < 0.25 on univariate analyses were included in multivariate analysis (Cox proportional hazards model). *P*< 0.05 was considered statistically significant. All statistical analyses were conducted using SPSS 17.0 (SPSS 17.0 for Windows, SPSS, Chicago, III). GKR: gamma knife radiosurgery; PVTT: portal vein tumor thrombosis; PV: portal vein; HBV:

hepatitis B virus; HCV: hepatitis C virus; ECOG: Eastern Cooperative Oncology Group; PST: Performance Status; AFP: alpha fetal protein.
